# Impact of the Endocardium in a Parameter Optimization to Solve the Inverse Problem of Electrocardiography

**DOI:** 10.3389/fphys.2018.01946

**Published:** 2019-01-22

**Authors:** Gwladys Ravon, Yves Coudière, Mark Potse, Rémi Dubois

**Affiliations:** ^1^IHU Liryc, Electrophysiology and Heart Modeling Institute, Pessac, France; ^2^Univ Bordeaux, CRCTB, U1045, Bordeaux, France; ^3^INSERM, CRCTB, U1045, Bordeaux, France; ^4^Carmen Research Team, Inria, Bordeaux, France; ^5^Univ Bordeaux, IMB UMR 5251, Talence, France

**Keywords:** ECGI, endocardium, parameter optimization, gradient descent method, Mitchell-Schaeffer, endo-epicardial gradients

## Abstract

Electrocardiographic imaging aims at reconstructing cardiac electrical events from electrical signals measured on the body surface. The most common approach relies on the inverse solution of the Laplace equation in the torso to reconstruct epicardial potential maps from body surface potential maps. Here we apply a method based on a parameter identification problem to reconstruct both activation and repolarization times. From an ansatz of action potential, based on the Mitchell-Schaeffer ionic model, we compute body surface potential signals. The inverse problem is reduced to the identification of the parameters of the Mitchell-Schaeffer model. We investigate whether solving the inverse problem with the endocardium improves the results or not. We solved the parameter identification problem on two different meshes: one with only the epicardium, and one with both the epicardium and the endocardium. We compared the results on both the heart (activation and repolarization times) and the torso. The comparison was done on validation data of sinus rhythm and ventricular pacing. We found similar results with both meshes in 6 cases out of 7: the presence of the endocardium slightly improved the activation times. This was the most visible on a sinus beat, leading to the conclusion that inclusion of the endocardium would be useful in situations where endo-epicardial gradients in activation or repolarization times play an important role.

## 1. Introduction

Electrocardiographic imaging aims at reconstructing cardiac electrical events from electrical signals measured on the body surface. The most common approach relies on the inverse solution of the Laplace equation in the torso to reconstruct epicardial potential maps from the body surface electrical potential maps (BSPM) (Wang and Rudy, 2006). This technique requires a regularization strategy to deal with the ill-posedness of the problem, for example Tikhonov regularization. However, as this regularization is applied to potential patterns, it suppresses the steep voltage gradients that characterize activation wavefronts. This leads to prominent errors such as artefactual block lines in the reconstructed activation map (Duchateau et al., 2017; Ravon et al., 2017).

Other methods have been designed to reconstruct directly the activation times (van Oosterom and Oostendorp, 1992; Liu et al., 2006). While Liu et al. (2006) look for the three-dimensional activation sequence in the ventricular muscle, van Oosterom and Oostendorp (1992) reconstruct activation on both the epicardium and the endocardium. van Dam et al. (2009) proposed a method that solved both the activation and the repolarization. Based on an equivalent double layer model, it updates activation and repolarization times alternatingly. Ghodrati et al. (2006) developed two methods to reconstruct epicardial information. One optimizes the position of the depolarization front at each time. The second reconstructs epicardial potentials with a regularization term based on the estimation of the wavefront behavior. These approaches still rely on a Tikhonov-like regularization technique. Recently, studies that reconstruct both the activation and the recovery, with a novel regularization technique, have been published (Cluitmans et al., 2017, 2018). The regularization is done through an electrophysiological input and the potentials on the torso are sparsely represented to deal with the ill-posedness of the problem. Others used a probabilistic approach to find parameters (Rahimi et al., 2016; Dhamala et al., 2018). The former used the two-variable Aliev-Panfilov model (Aliev and Panfilov, 1996) to model the AP. Their aim was to probabilistically personalize a model parameter using machine learning methods. The estimation was made on a whole-heart 3D model, from BSPMs or extracellular potentials. In the latter the parameters of the model are assumed and the behavior of the wavefront is optimized. The same group worked on regularizing both the spatial and the temporal propagation of action potential (Wang et al., 2010). The method relies on a two-variable propagation model with fixed parameters in a volumetric myocardium. It was then improved in Ghimire et al. (2017). Note that in these studies constraints in the spatial distribution are considered.

In a previous study (Ravon et al., 2017) we introduced a new technique that aims at recovering directly both the activation and repolarization maps on the epicardium. The general idea consists in looking for an ansatz of an action potential (AP) under the form of a function *v*(*P*; *t*) parameterized by a small number of parameters *P*, e.g., less than three. The upstroke of this AP is supposed to be at *t* = 0. From the knowledge of the activation times τ (*x*) on the heart, we can map the AP to a space- and time-dependent function *V*_m_(*t, x*) = *v*(*P*; *t* − τ). In addition, the parameters *P* may have space-dependent values distributed on the surface, which enriches the model, but increases the number of unknown parameters. Then this transmembrane voltage function *V*_m_(*t, x*) is projected to body surface potential signals. The method searches for the parameters *P* and activation map τ that realize the best fit to the target body surface signals on a given time interval. It amounts to solving a nonlinear least squares parameter identification problem with a small number of (possibly distributed) parameters. We previously represented the action potential as the product of two logistic functions, as proposed by Van Oosterom and Jacquemet (2005). The final parameter identification problem (Ravon et al., 2017) consisted of identifying three distributed parameters, given the BSPM of a complete ventricular activation and repolarization sequence (i.e., a QRST waveform). This method was demonstrated to give a better range of activation times (ATs) and a smoother AT distribution than a solution based on the Laplace equation with Tikhonov regularization of order zero. However, it only reconstructed APs on the epicardium. In general, large and physiologically very relevant differences in AT and repolarization time (RT) can exist across the wall. Therefore, in this study we investigated whether including the endocardium improves the results.

To this aim, we tested our method on *in silico* data with and without important transmural gradients. The parameter identification problem was solved either on the epicardium only, or on both the epicardium and endocardium. We found that the quality of the reconstructed activation and repolarization maps (in terms of correlation coefficients) was similar when transmural gradients were small, but that inclusion of the endocardium improved the solution in a case where these gradients were important.

As compared to Ravon et al. (2017), we also changed the representation of the AP from the product of two logistic functions to the solution of the two-variable ionic model of Mitchell and Schaeffer (2003), to have a more relevant AP shape without increasing the number of parameters.

We resorted to a discretize-then-optimize strategy: we first set the direct problem that maps the parameters *P* and activation map τ to the voltage *V*_m_(*t, x*), and then to the BSPM ϕ_T_. This problem was discretized using triangulated surfaces. The parameters were identified in the discrete problem using a gradient descent method on a discrete least squares cost function.

## 2. Materials and Methods

### 2.1. Mapping the Parameters to the Transmembrane Voltage

The parameterization was based on the two-current model proposed by Mitchell and Schaeffer (2003). This model describes the dynamics of two functions: the voltage *v* and an auxiliary variable *h*. Both quantities are dimensionless and scaled between 0 and 1, and solve the following ordinary differential equations:

(1) $$\begin{array}{l} v^{\prime }=\frac{hv^{2}\left(1-v\right)}{\tau_{\text{in}}}-\frac{v}{\tau_{\text{out}}}, \end{array}$$

(2) $$\begin{array}{l} h^{\prime }=\frac{1-h}{\tau_{\text{open}}}\;\text{if }v<v_{\text{gate}},\;\text{and}\;h^{\prime }=\frac{-h}{\tau_{\text{close}}}\;\text{if}v>v_{\text{gate}}. \end{array}$$

**Figure 1:**
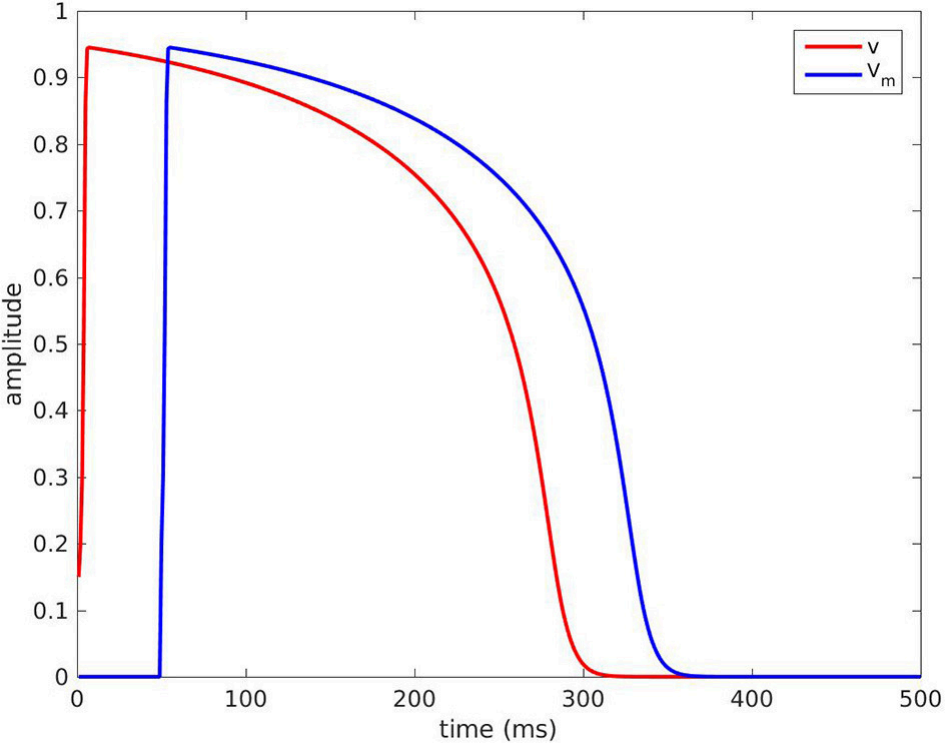
Red curve: voltage *v*(*P, t*) with the default parameters *P*. Blue curve: TMP *V*_m_(*t*) = *v*(*P*; *t* − τ) with τ = 50 ms.

The five parameters were originally chosen as (Mitchell and Schaeffer, 2003): τ_in_ = 0.3 ms, τ_out_ = 6 ms, τ_open_ = 120 ms, τ_close_ = 150 ms, and *v*_gate_ = 0.13. The steady state for this model is (*v, h*) = (0, 1). The voltage *v* takes the shape of an AP if we set the initial condition as (*v*(0), *h*(0)) = (0.15, 1), see the red curve in Figure 1.

The function *v*(*t*) defined for *t* ≥ 0 as the solution of the initial value problem (1)-(2) with (*v*(0), *h*(0)) = (0.15, 1) was completed by 0 for *t* < 0. It was our ansatz of an AP, denoted by *v*(*P*; *t*) for *t* ∈ ℝ, and in general *P* = {τ_in_, τ_out_, τ_open_, τ_close_, *v*_gate_}. For instance, the blue curve in Figure 1 is the graph of *v*(*P, t* − τ) for an activation time τ = 50 ms and the default values for *P* stated above.

In practice, the parameters τ_in_ and τ_open_ define the upstroke of the AP, and were fixed with their default values τ_in_ = 0.3 ms and τ_open_ = 120 ms. Similarly, the parameter *v*_gate_ defines the excitability threshold and was fixed at *v*_gate_ = 0.13. Hence, only the parameters τ_out_ and τ_close_ were searched as unknown parameters, because they are directly related to the AP duration. τ_close_ can be seen as the plateau phase duration whereas τ_out_ is linked to the speed of the repolarization. τ_out_ also has a small impact on the amplitude of the voltage *v*.

In addition, we rescaled the voltage *v* by a factor A, so as to fit the scaling of the measured BSPM. Hence, we considered the mapping

(3) $$\begin{array}{l} P:=\left(A,\underset{P}{\underset{︸}{\tau_{\text{out}},\tau_{\text{close}}}},\tau \right)\in {\mathbb{R}}^{4}\mapsto V_{\text{m}}\left(x,t\right)=Av\left(P,t-\tau \right). \end{array}$$

The parameter τ was distributed on the heart surface by the design of the method. Meanwhile, the parameters A, τ_out_, and τ_close_ may be constant or distributed. Since AP duration varies across the heart surface, we would rather consider varying distributed parameters τ_out_ and τ_close_.

### 2.2. Projecting the Transmembrane Voltage to the Body Surface Potential Map

Afterwards, we mapped the transmembrane voltage *V*_m_(*x, t*) to extracellular potentials ϕe(*x, t*) as in Potse et al. (2009):

(4) $$\begin{array}{l} V_{\text{m}}\left(x,t\right)\mapsto \phi \text{e}\left(x,t\right)=\bar{V_{\text{m}}}\left(t\right)-V_{\text{m}}\left(x,t\right), \end{array}$$

where $\bar{V_{\text{m}}}\left(t\right)$ was a fixed spatial average of *V*_m_(*x, t*), $\bar{V_{\text{m}}}\left(t\right)=\frac{1}{\left|S\right|}\underset{S}{\int }V_{\text{m}}\left(x,t\right)ds\left(x\right)$ where *S* is the heart surface (epicardium only, or epicardium and endocardium). The rationale of the formula is a rewriting of the bidomain model coupled with the hypothesis that conductivity tensor fields in both extra- and intra-cellular domains are homogeneous and isotropic. Here the ratio of conductivities was hidden in the factor A. Finally, we projected the extracellular potentials ϕe(*x, t*) to the body surface potentials ϕ_T_(*y, t*) for any point *y* on the torso surface as follows:

(5) $$\begin{array}{l} \phi \text{e}\left(x,t\right)\mapsto \phi_{\text{T}}\left(y,t\right)=\underset{S}{\int }\frac{1}{4\pi ||x-y||}\phi \text{e}\left(x,t\right)ds\left(x\right), \end{array}$$

This amounted to approximating the solution of the Laplace equation outside the heart domain, assuming it is an infinite homogeneous medium (Malmivuo and Plonsey, 1995; Macfarlane et al., 2010).

### 2.3. Discrete Surfaces and Approximations

**Figure 2:**
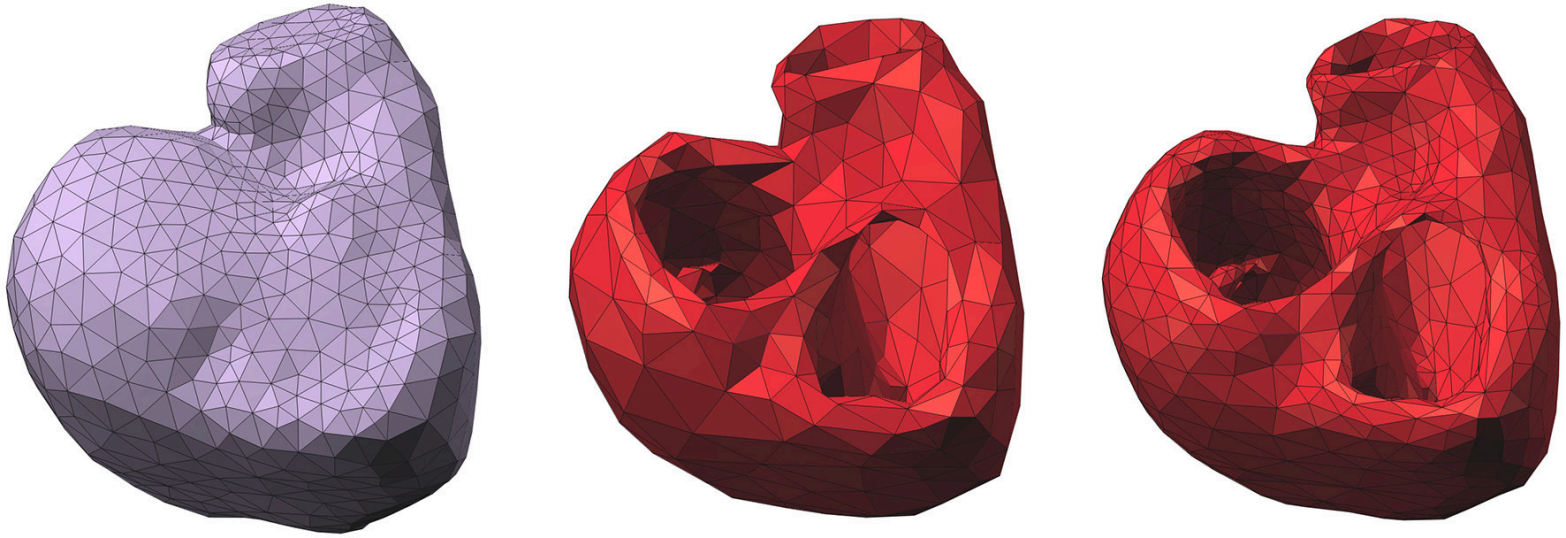
**Left**: epicardium-only mesh (*Mesh1*); **middle**: endocardial and epicardial mesh (*Mesh2*); **right**: refined mesh of *Mesh2*. Right posterior oblique view.

In practice, the endocardial and epicardial surfaces were discretized by two separate triangular meshes (Figure 2) with vertices denoted by (*x*_*i*_)_*i* = 1…*N*_*H*__. The endocardial surface included the surface of the free wall and the septum (Figure 2, middle).

For the sake of computational simplicity, the mappings (4) and (5) were replaced by their discrete counterparts:

(6) $$\begin{array}{l} \phi \text{e}\left(x_{i},t\right)=\frac{1}{N_{H}}\overset{N_{H}}{\underset{k=1}{\sum }}V_{\text{m}}\left(x_{k},t\right)-V_{\text{m}}\left(x_{i},t\right),\;\\ \;\phi_{\text{T}}\left(y,t\right)=\overset{N_{H}}{\underset{i=1}{\sum }}\frac{1}{4\pi ||x_{i}-y||}\phi \text{e}\left(x_{i},t\right), \end{array}$$

where *V*_m_(*x*_*i*_, *t*) was given by the mapping (3) for given parameters A ∈ ℝ, ${\left(\tau_{\text{out}}\left(x_{i}\right)\right)}_{i}\in {\mathbb{R}}^{N_{H}}$, ${\left(\tau_{\text{close}}\left(x_{i}\right)\right)}_{i}\in {\mathbb{R}}^{N_{H}}$ and ${\left(\tau \left(x_{i}\right)\right)}_{i}\in {\mathbb{R}}^{N_{H}}$. Hence there are 1 + 3*N*_*H*_ parameters to be identified.

### 2.4. The Parameter Identification Problem

We looked for the parameter set $P=\left(A,\tau_{\text{out}},\tau_{\text{close}},\tau \right)\in {\mathbb{R}}^{1+3N_{H}}$ that minimized the least squares error

(7) $$\begin{array}{l} J\left(P\right)=\frac{1}{2}\overset{T_{\text{max}}}{\underset{k=1}{\sum }}\overset{N_{T}}{\underset{j=1}{\sum }}|\left(\phi_{\text{T}}\left(y_{j},t_{k}\right)-\bar{\phi_{\text{T}}\left(t_{k}\right)}\right)-\left(\phi^{⋆}\left(y_{j},t_{k}\right)-\bar{\phi^{⋆}\left(t_{k}\right)}\right){|}^{2}, \end{array}$$

where (*y*_*j*_)_*j* = 1…*N*_*T*__ were the *N*_*T*_ electrode locations on the body surface, (*t*_*k*_)_*k* = 1…*T*_max__ was the time sequence of interest, $\left(\phi^{⋆}\left(y_{j},t_{k}\right)\right)$ were the measured BSPMs, and (ϕ_T_(*y*_*j*_, *t*_*k*_)) were the BSPMs computed according to equations (6). For each time *t*_*k*_, the spatial averages $\bar{\phi_{\text{T}}\left(t_{k}\right)}$ and $\bar{\phi^{⋆}\left(t_{k}\right)}$ were defined by $\bar{\phi_{\text{T}}\left(t_{k}\right)}=\frac{1}{N_{T}}\overset{N_{T}}{\underset{j=1}{\sum }}\phi_{\text{T}}\left(y_{j},t_{k}\right)$ and $\bar{\phi^{⋆}\left(t_{k}\right)}=\frac{1}{N_{T}}\overset{N_{T}}{\underset{j=1}{\sum }}\phi^{⋆}\left(y_{j},t_{k}\right)$. Potentials are given up to a constant. This constant can be a reference electrode on the torso, the WCT or the mean of all the electrodes. We chose the mean. As Wilson's Central Terminal it was also a way to reduce noise. Moreover, it rescaled the data around their mean value.

The total number of data elements is finally *T*_max_*N*_*T*_, which may be compared to the number of unknown parameters 1 + 3*N*_*H*_. This nonlinear least squares problem was solved by the gradient descent method with the RMSprop update (Tieleman and Hinton, 2012). This is an adaptive learning rate method: at each iteration, the update reads:

(8) $$\begin{array}{l} \kappa :=\gamma \kappa +\left(1-\gamma \right)\nabla_{P}J⊗\nabla_{P}J\;\text{in }{\mathbb{R}}^{1+3N_{H}}, \end{array}$$

(9) $$\begin{array}{l} P:=P-\eta \nabla_{P}J⊘\left(\kappa^{{}^{\circ}1/2}+10^{-7}\right)\;\text{in }{\mathbb{R}}^{1+3N_{H}}, \end{array}$$

with $\kappa \in {\mathbb{R}}^{1+3N_{H}}$ an intermediate variable, η ∈ ℝ the learning rate and γ = 0.9. The learning rate was not fixed, an optimal value for η was chosen at each iteration in the range [10^−5^, 10^2^]. In equations 8 and 9 the operators ⊗, ⊘, and ° denote the Hadamard product, division, and power, respectively. The gradient of the cost function *J* with respect to the unknown parameters P was calculated analytically.

For the gradient descent method, an initial guess was required. We arbitrarily chose A = 10, the default values τ_out, *i*_ = 6ms and τ_close, *i*_ = 150ms for all *i*, and τ_*i*_ constant τ_*i*_ = τ_0_ ∈ ℝ. Since the initialization was the same for all the nodes, the initial torso potentials were zero. The optimization ended when the cost function *J* and its gradient remained constant. The code was in Matlab and not parallel. Computational time was quite long and similar for all the cases, namely about one day. A more flexible stopping criterion and parallelism would reduce computational time.

### 2.5. Validation Data

**Figure 3:**
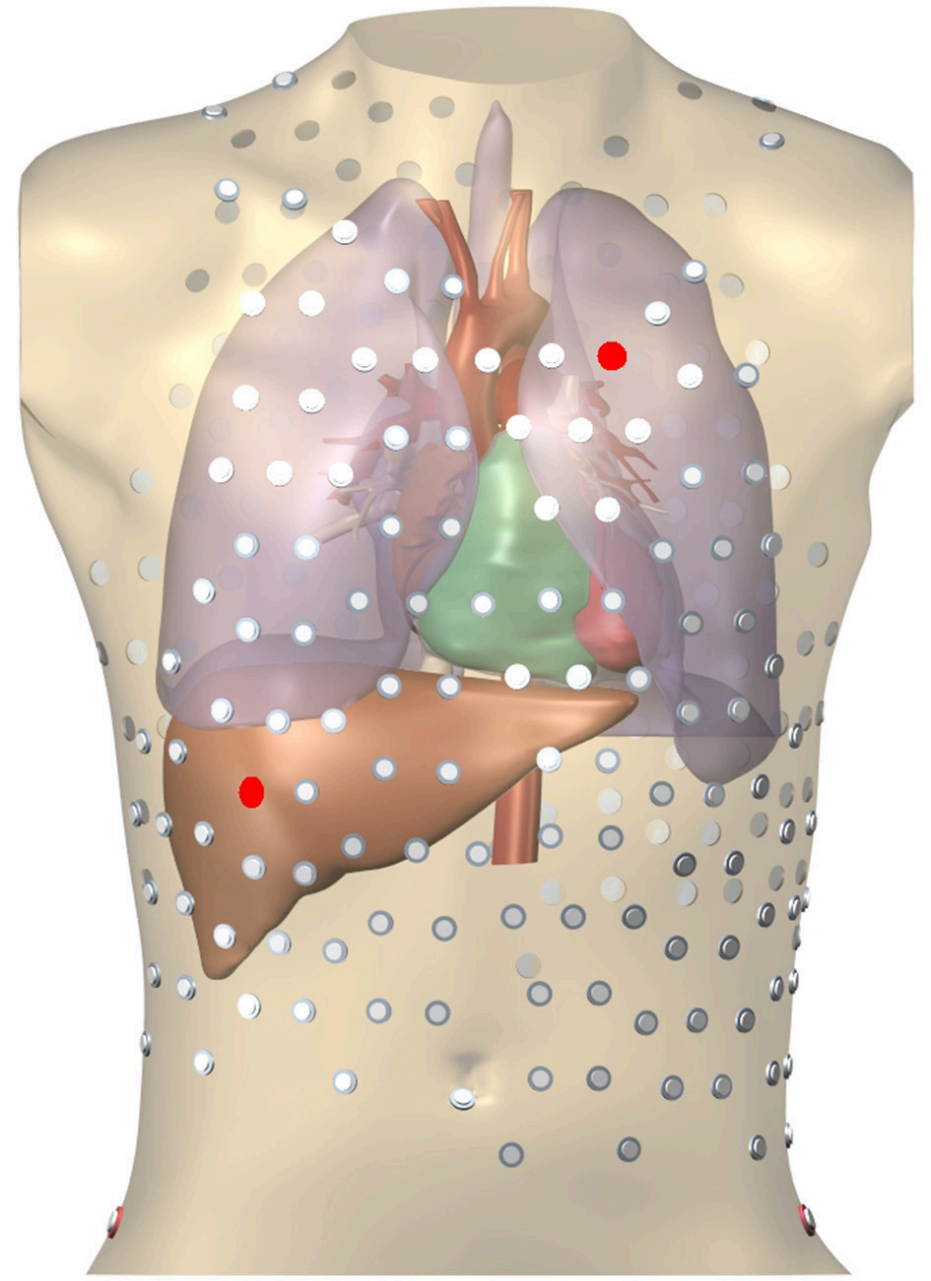
Heart-torso mesh used for the computation of validation data. The 252-electrode body surface mapping set is shown. Red electrodes mark two locations used in Figure 8.

In order to create testing data, simulations were run on an anatomically realistic 3D geometry of the torso, including heart, blood vessels, lungs, and skeletal muscle (Figure 3). Each organ had its own conductivity. Propagating AP were generated using a monodomain reaction-diffusion model with a TNNP membrane model (Ten Tusscher et al., 2004) on an anisotropic heart model at 0.2 mm resolution. To compute ϕ_T_ the computed transmembrane current density in the myocardium was projected on an inhomogeneous heart-torso model with anisotropic skeletal muscle layer at 1 mm resolution and the potential field ϕ_T_ was found by solving an anisotropic Laplace problem using a finite-difference method (Potse, 2018). Boundary conditions did not match between the monodomain model and the Laplace equation. This approach leads to slightly different extracellular potentials within a few hundred micrometers from the surface only (Potse et al., 2006). All simulations were performed with a recent version of the Propag-5 software (Krause et al., 2012) on a BullX cluster machine.

We had access to the activation times on the epicardium and the endocardium (named reference ATs in the following). Repolarization times were computed from extra-cellular potentials as the time with highest positive slope during the repolarization phase.

## 3. Results

On the same model anatomy, seven different simulations were run: one sinus rhythm (SR) and six different pacing cases. The description of the cases can be found in Table 1. For all the cases, we solved the parameter identification problem on the epicardium-only mesh (*Mesh1*) and on the epicardium and endocardium mesh (*Mesh2*). *Mesh1* and *Mesh2* had 641 and 534 vertices respectively. We will describe the results in detail for two cases: right-ventricular pacing and sinus rhythm.

**Table 1:** Description of the 7 cases.

**Case**	**Description**
1	Epicardial ventricular pacing
2	Sinus rhythm
3	Endocardial ventricular pacing
4	Epicardial ventricular pacing (near apex)
5	Endocardial ventricular pacing (near apex)
6	Pacing on the basis of the pulmonary vein
7	Pacing on the septum, halfway up to the right ventricle

### 3.1. Epicardial Ventricular Pacing

**Figure 4:**
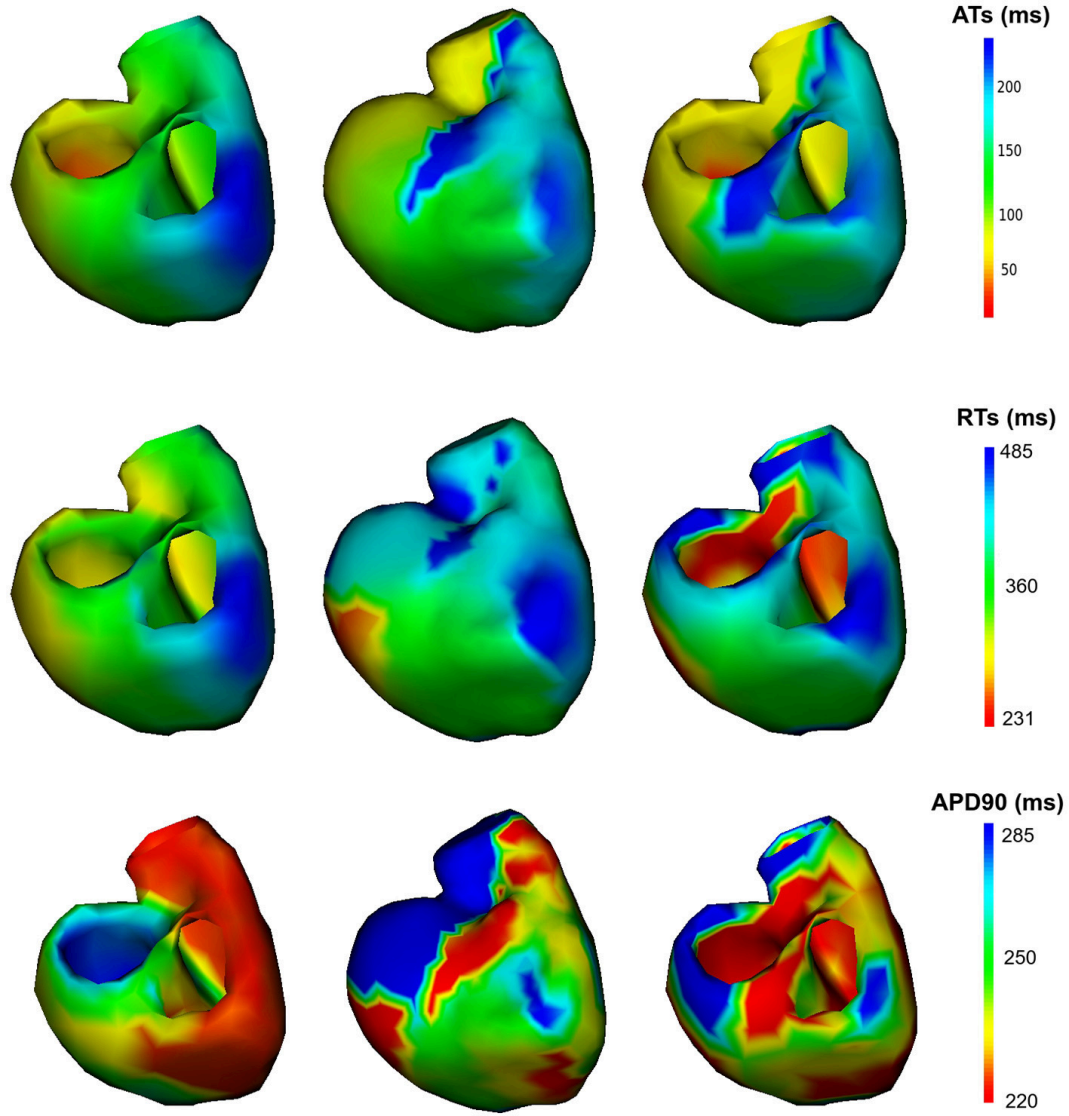
Activation, repolarization and APD90 maps of the RV pacing case. **Left**: reference ATs; **middle**: optimized ATs from *Mesh1*; **right**: optimized ATs from *Mesh2*. Right posterior oblique view.

**Figure 5:**
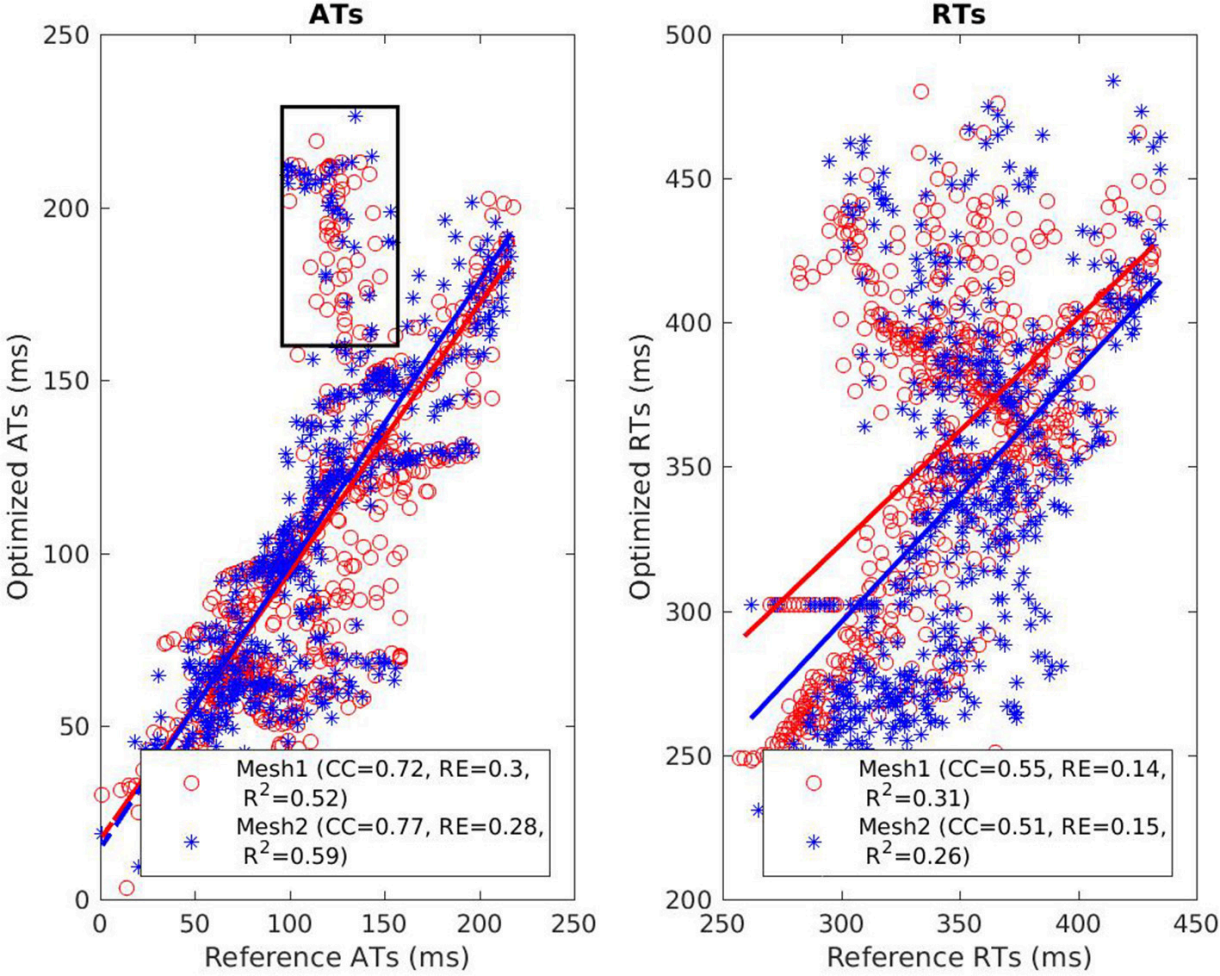
Scatter plot of the ATs **(left)** and RTs **(right)** for the RV pacing case. For each point, the *x* coordinate is the reference AT (resp. RT) and the *y* coordinate is the corresponding reconstructed AT (resp. RT). The dashed lines represent the linear fitting. The black box on the left exhibits ATs that were badly reconstructed with both meshes.

The reconstructed activation maps in case of right-ventricular pacing were of the same quality on both meshes. In particular the late ATs were not well reconstructed in both cases (first row, dark blue part in Figure 4). The correlation coefficient (CC) and relative error (RE) between ATs were close for both meshes, about 0.7 and 0.3 respectively. However, Figure 5 shows that a part of the reference ATs between 120 and 160 ms was less well reconstructed with *Mesh1* than with *Mesh2*. For both meshes some reference ATs between 100 and 150 ms were not well reconstructed (Figure 5, left, black box). These points were located between the two valves, where the reconstruction is more difficult. The pacing site was better localized with *Mesh1* (11.4 mm from the actual position, geodesic distance) than with *Mesh2* (16 mm), as shown in **Figure 12**. For *Mesh2* we also calculated CC for the points on the epicardium (CC = 0.72) and on the endocardium (CC = 0.77). With the endocardium we did not improve the accuracy on the epicardium compared to the results with the epicardium only.

**Figure 6:**
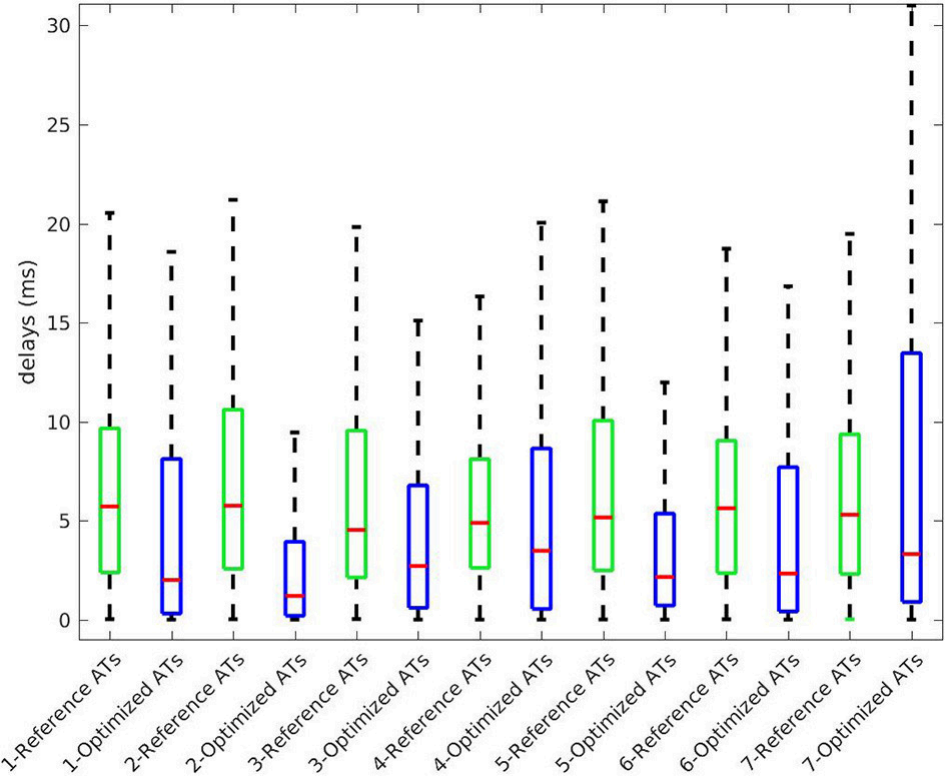
Delays in ATs between endocardium and epicardium for the 7 cases. Box plot represent the median and the first and third quartiles. Whiskers represent the extreme values. Optimized ATs are AT given by the inverse method with *Mesh2*.

The benefit of considering the endocardium was to look for gradients of depolarization between the endocardium and epicardium. For each point on the epicardium, we selected the closest point on the endocardium and computed the delay in the activation. Figure 6 presents box plot of these delays for the 7 cases. Delays existed in the reference ATs (first box) and the delays we obtained were smaller on average. We also obtained large delays (more than 20 ms and up to 135) that were not consistent with the actual ones.

On both meshes, the quality of repolarization maps was less good than the activation maps (Figure 4, second row). The CC was slightly better with *Mesh1* (0.55 vs. 0.51). It was highlighted on the scatter plot, especially for the earlier RTs (Figure 5, right).

**Figure 7:**
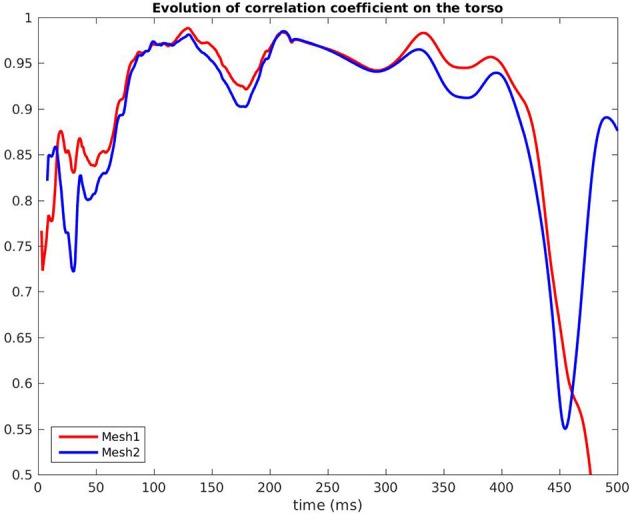
Correlation coefficient of the BSPM, RV pacing case.

**Figure 8:**
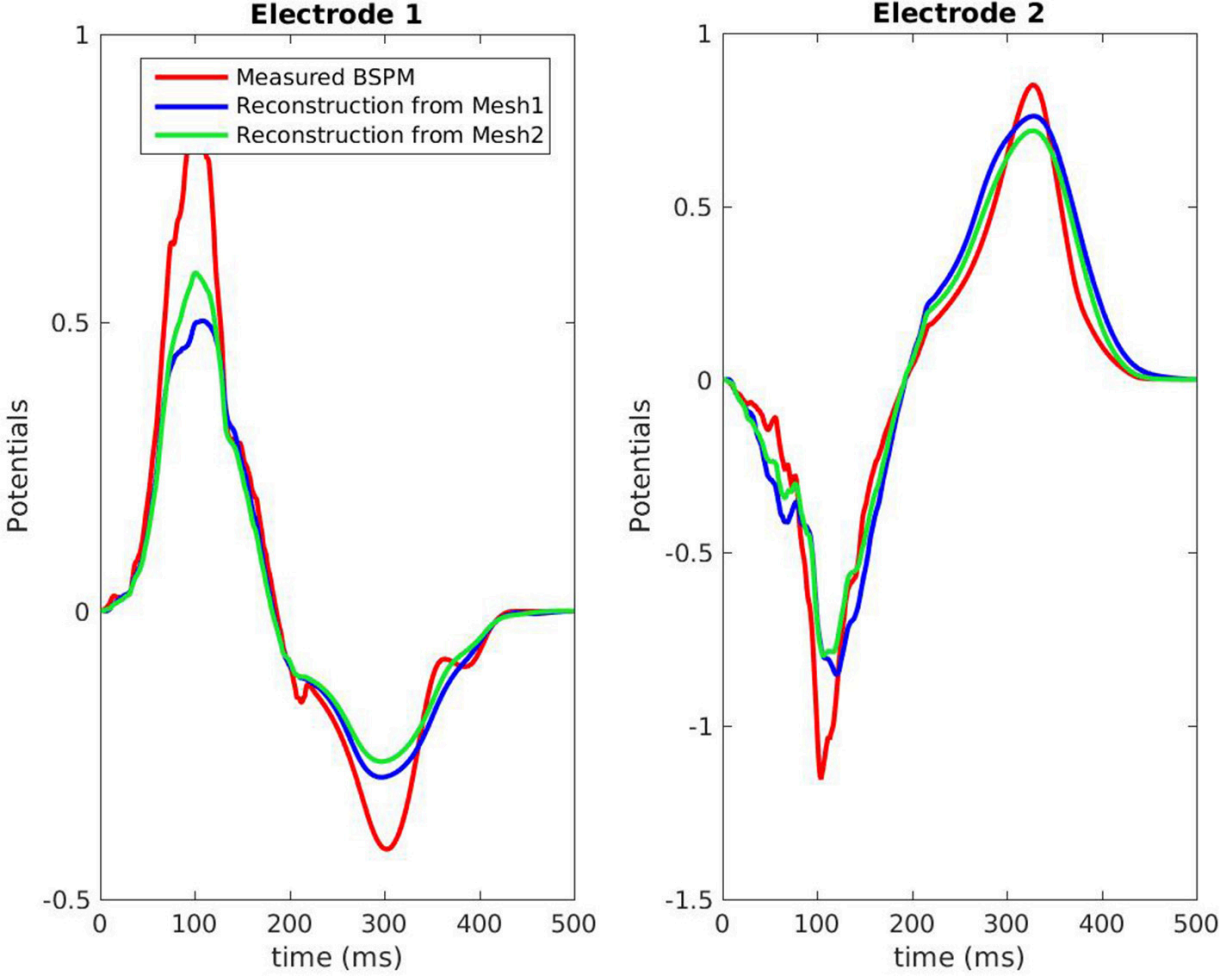
Potentials on the torso, RV pacing case. The locations of the 2 electrodes are marked in red in Figure 3.

**Figure 9:**
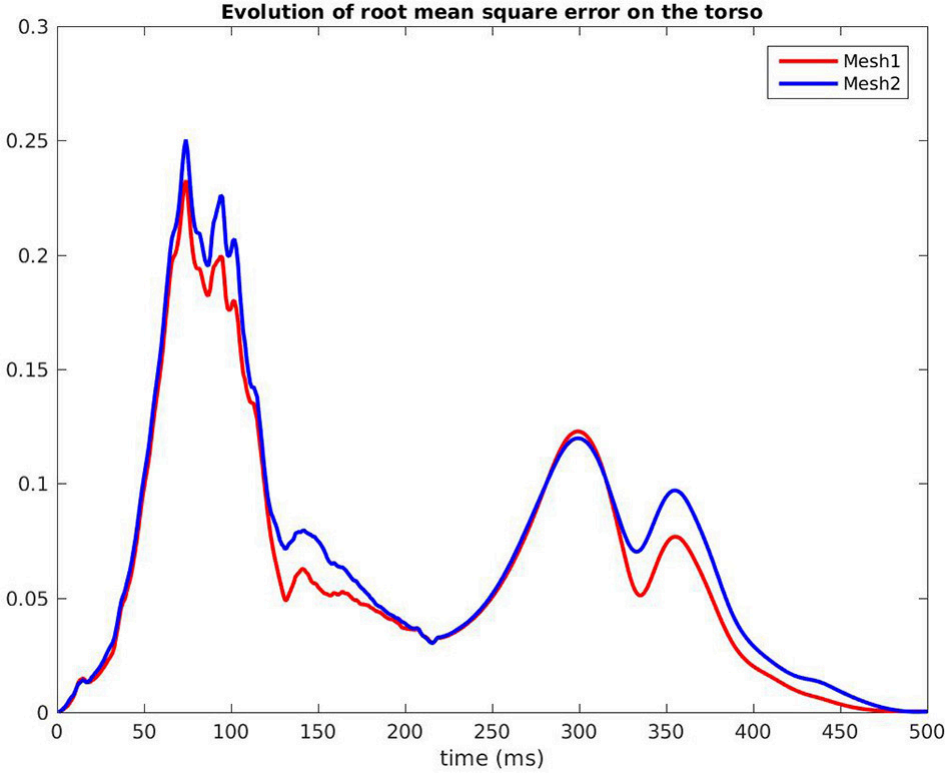
Root mean square error of the BSPM, RV pacing case.

Figure 7 shows the evolution in time of the CC between the measured BSPM and the reconstructed ones. Reconstructed torso potentials were computed from equation (3), (4), and (5) with the optimized parameters and the corresponding mesh *Mesh1* or *Mesh2*. On both meshes, the behavior was similar: at the beginning and the end of the simulation the reconstruction was less accurate. As shown by Figure 8, after 400 ms, measured and reconstructed BSPMs are close to zero, which explained that the CC dropped. On average, the CC was 0.88 with *Mesh1*, and 0.9 with *Mesh2*. On both electrodes, depolarization, and repolarization phases were quite well fitted for the two meshes. There were just slight differences between the reconstructed BSPMs. We also calculated the root mean square error (RMSE) between the measured BSPMs and the reconstructed ones (Figure 9). Two peaks can be seen: one corresponding to the depolarization phase and the second to the repolarization phase. They were mainly due to the amplitude: the optimized amplitude did not allow to fit the signals on all the electrodes (Figure 8). RMSE was similar for the 2 meshes.

### 3.2. Sinus Rhythm

**Figure 10:**
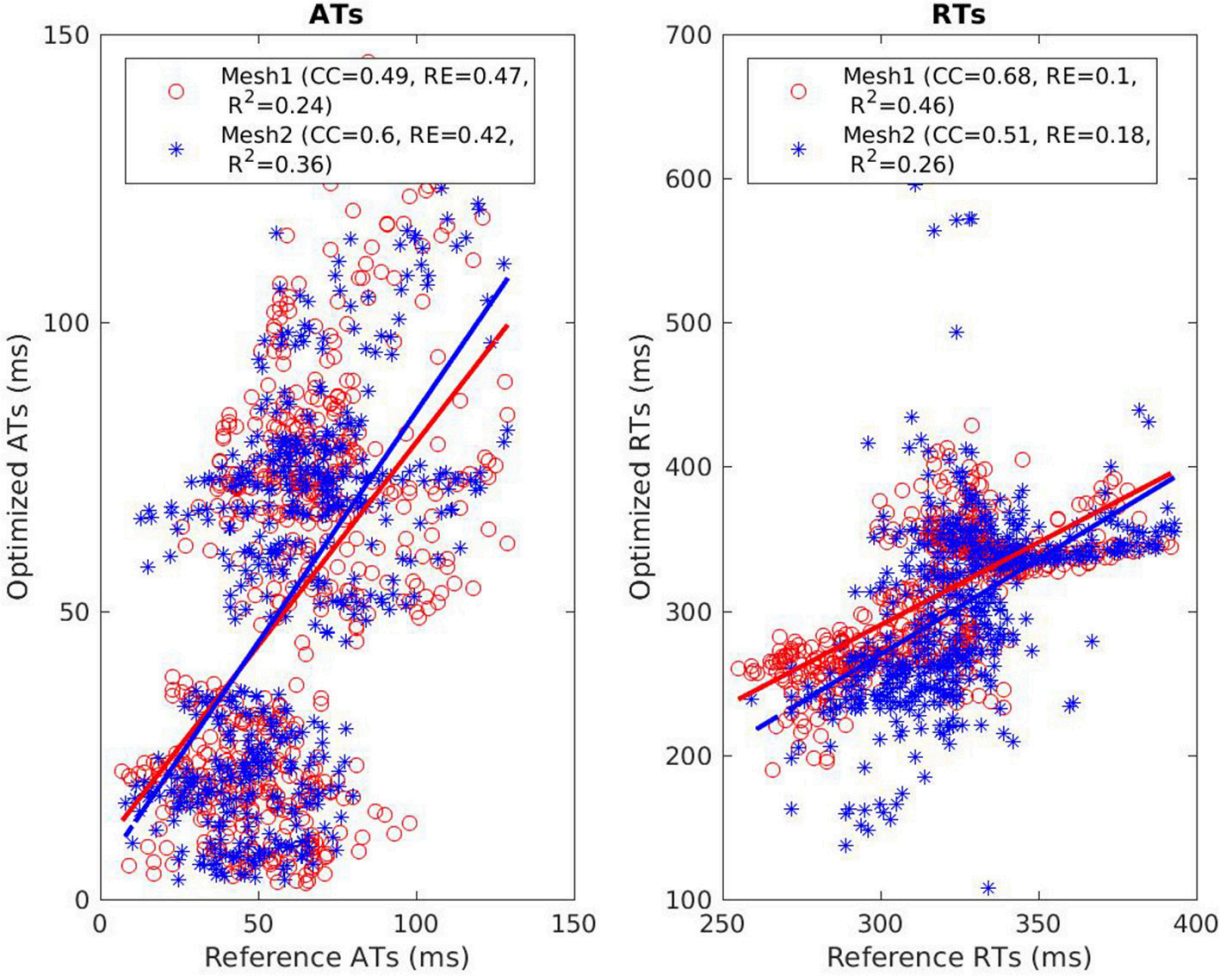
Scatter plot of the ATs **(left)** and RTs **(right)** for the SR case. For each point, the *x* coordinate is the reference AT (resp. RT) and the *y* coordinate is the corresponding reconstructed AT (resp. RT). The dashed lines represent the linear fitting.

It is well known that the QRS duration is shorter in sinus beat than in a paced beat. Moreover, there were multiple breakthroughs in the myocardium. For these reasons it was harder to obtain a satisfying reconstruction than in the pacing cases. For both meshes the reconstructed total activation time was longer than the actual. The CC and RE were better with the endocardium than without, but still not as good as in the pacing cases (Figure 10, left). For *Mesh2* we also calculated CC for the points on the epicardium (CC = 0.64) and on the endocardium (CC = 0.57). With the endocardium we improved the accuracy on the epicardium (CC = 0.64) compared to the results with *Mesh1* (CC = 0.49).

**Figure 11:**
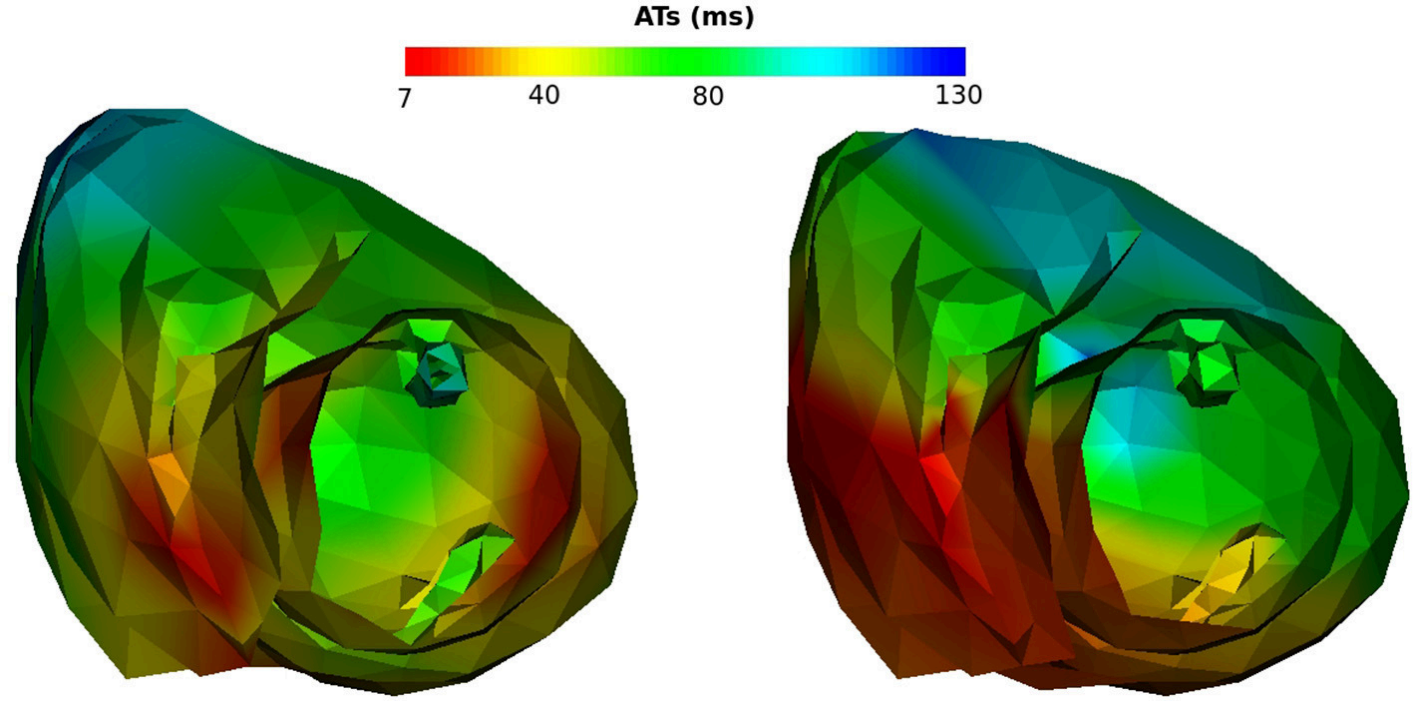
Activation maps on *Mesh2*, SR case. **Left**: reference activation map, **right**: reconstructed activation map.

We also looked at the delays between endocardium and epicardium (Figure 6). These were similar on the reference ATs for the SR and RV pacing case (first and third boxes). Since the total activation time (TAT) is smaller in a sinus beat, the relative values of these gradients to the TAT were more important than in RV pacing. We reconstructed different delays for this two cases. The delays were not reconstructed as well for the SR as for the pacing cases. Indeed as shown in Figure 11, there was a gradient of activation on the left ventricular free wall that we did not recover. Similarly there were delays in the activation of the septum that we did not reconstruct.

CC and RE for the repolarization times were better with *Mesh1*: 0.51 and 0.18 respectively with the endocardium and 0.68 and 0.1 without (Figure 10, right). Indeed with the endocardium the range of RTs was much larger, from *t* = 108 ms to *t* = 628 ms, whereas the actual range was from *t* = 259 ms to *t* = 393 ms.

Finally we compared the signals on the torso. As in the pacing case, CC and RMSE evolved in the same way for both meshes, with close values over time. In both cases the CC dropped after 350 ms because reconstructed T waves sometimes ended later than the real ones. In the simulation the heart was almost at rest after 350 ms, which was not the case with our optimized parameters. On average, the CC was 0.83 with *Mesh1*, and 0.87 with *Mesh2*.

#### 3.2.1. Sensitivity to the Initialization

In order to test if the method was sensitive to the initialization, we solved the inverse problem with two other triplets. The results we previously presented were obtained from the triplet (τ_*i*_, τ_out, *i*_, τ_close, *i*_) = (60, 6, 150). The second and third triplets were (75, 5, 130) and (75, 6, 15) respectively. The results are presented in Table 2. The three initializations ended with very close results: CC for ATs and RTs were in the same range, as well as for the BSPM. Moreover, for the three triplets, the method gave a better accuracy of the ATs with *Mesh2*, while RTs were better reconstructed with *Mesh1*. Changing the initial ATs did not improve the accuracy on the reconstructed ATs. Finally, the reconstructed torso potentials were very close to each other for the three initializations (CC between 0.83 and 0.9). Especially, the QRS complex and the T wave were fitted in the same way.

**Table 2:** Comparison of different initializations for the sinus rhythm case. Each triplet is of the form (τ_*i*_, τ_out, *i*_, τ_close, *i*_).

		**Heart**		**Torso**	
		**ATs**	**RTs**	**BSPM**	**Reduction of J (%)**
**Initialization**	**Mesh**	**CC**	**CC**	**CC**	
(60, 6, 150)	*Mesh1*	0.49	0.68	0.83 ± 0.25	87
	*Mesh2*	0.6	0.51	0.87 ± 0.17	83
(75, 5, 130)	*Mesh1*	0.44	0.65	0.9 ± 0.12	83
	*Mesh2*	0.51	0.54	0.87 ± 0.16	81
(75, 6, 150)	*Mesh1*	0.48	0.65	0.84 ± 0.26	86
	*Mesh2*	0.59	0.57	0.89 ± 0.12	82

### 3.3. All the Cases

We present the results for all the cases in Table 3. A box plot representation can be found on Supplementary Material, as well as activation, repolarization, and APD90 maps for all the cases. In cases 4 and 6 CC of ATs were better with *Mesh2*. In all others cases, CC were similar for both meshes. In all cases, solving the inverse problem with *Mesh2* gave at least as accurate ATs on the epicardium as with *Mesh1*. Optimized RTs were better with *Mesh2* in only 2 cases: pacing on the basis of the pulmonary vein (case 6) and pacing on the septum (case 7). Figure 6 shows the delays in activation. On average we reconstructed smaller delays in all cases.

**Table 3:** Results for the 7 cases.

		**Heart**						**Torso**		
		**ATs**			**RTs**			**BSPM**		
**Case**	**Mesh**	**CC**	**RE**	**CC epi**	**CC**	**RE**	**CC epi**	**CC**	**RMSE**	**Reduction of J (%)**
1	*Mesh1*	0.72	0.3		0.55	0.14		0.88 ± 0.19	0.06 ± 0.05	90
	*Mesh2*	0.77	0.28	0.72	0.51	0.15	0.5	0.9 ± 0.1	0.07 ± 0.06	86
2	*Mesh1*	0.49	0.47		0.68	0.1		0.83 ± 0.25	0.04 ± 0.04	87
	*Mesh2*	0.6	0.42	0.64	0.51	0.18	0.5	0.87 ± 0.17	0.05 ± 0.05	83
3	*Mesh1*	0.86	0.22		0.7	0.16		0.89 ± 0.17	0.15 ± 0.09	88
	*Mesh2*	0.85	0.23	0.89	0.61	0.19	0.69	0.86 ± 0.26	0.15 ± 0.1	87
4	*Mesh1*	0.67	0.32		0.75	0.14		0.84 ± 0.23	0.16 ± 0.13	78
	*Mesh2*	0.74	0.31	0.67	0.54	0.19	0.44	0.81 ± 0.24	0.16 ± 0.13	79
5	*Mesh1*	0.73	0.28		0.76	0.13		0.84 ± 0.26	0.12 ± 0.1	87
	*Mesh2*	0.72	0.29	0.72	0.7	0.14	0.75	0.85 ± 0.24	0.12 ± 0.1	87
6	*Mesh1*	0.66	0.35		0.67	0.17		0.74 ± 0.44	0.15 ± 0.14	74
	*Mesh2*	0.77	0.23	0.74	0.7	0.15	0.7	0.75 ± 0.47	0.14 ± 0.13	77
7	*Mesh1*	0.4	0.42		0.38	0.17		0.88 ± 0.13	0.05 ± 0.05	87
	*Mesh2*	0.45	0.41	0.43	0.57	0.13	0.48	0.58 ± 0.47	0.08 ± 0.06	89

*Case 1: epicardial ventricular pacing. Case 2: sinus rhythm. Case 3: endocardial ventricular pacing. Case 4: epicardial ventricular pacing (near apex). Case 5: endocardial ventricular pacing (near apex). Case 6: pacing on the basis of the pulmonary vein. Case 7: pacing on the septum*.

Concerning the reconstructed BSPMs, averaged CC and RMSE are given in Table 3. Except in case 7, the averaged CC were very similar for both meshes. They kept very close values over time. We observed the same behavior for the RMSE in all the cases. The lower averaged CC in case 7 with *Mesh2* was due to a shorter total activation time: late ATs were not well reconstructed.

A statistical *T*-test was performed on the CC for ATs, RTs, and BSPM. The resulting *p*-values were 0.5, 0.41, and 0.28 respectively, showing no significant differences between the two meshes.

**Figure 12:**
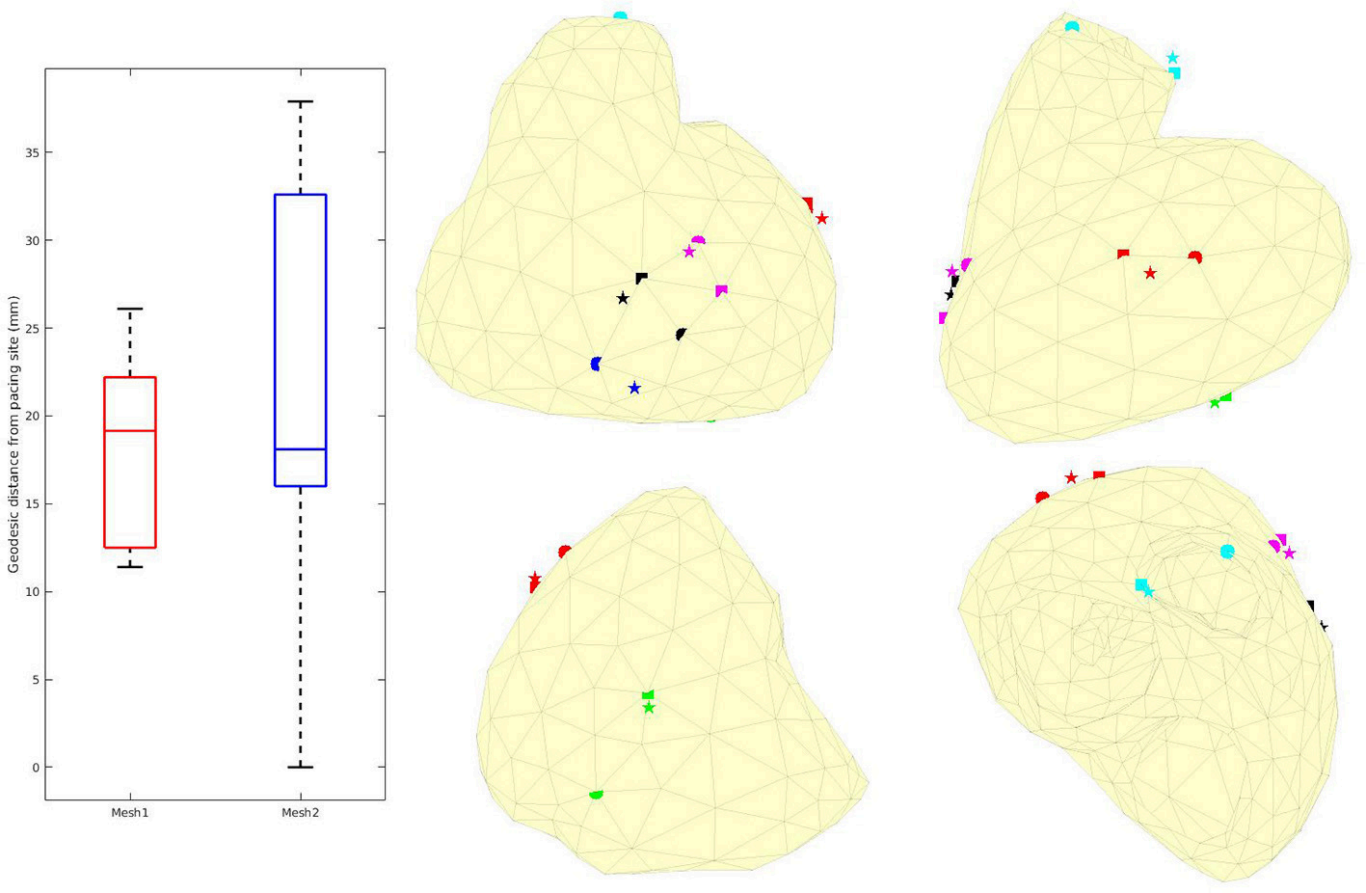
**Left**: Box plots of the geodesic distances between actual and identified pacing sites. **Middle and right**: actual (sphere), identified with *Mesh1* (pentagram) and *Mesh2* (square) pacing locations. Red: case 1, blue: case 3, green: case 4, black: case 5, cyan: case 6, magenta: case 7. We obtained a *p*-value of 0.74.

We computed the geodesic distance between the actual pacing site locations and the one given by the inverse solution for cases 1, 4, and 6 (epicardial pacing). For endocardial pacing (cases 3, 5, and 7) we computed the distance between actual and reconstructed breakthrough on the epicardium. From the optimization, the pacing site (or breakthrough) was identified as the mesh node with the earliest AT (resp. on the epicardium). We added a visual validation to exclude irrelevant, isolated, early ATs. Results can be found in Figure 12. In most of the cases, the distance was smaller with *Mesh1* than *Mesh2*. However, except for case 6, the identified site with *Mesh2* was a neighbor of the actual site. So the differences in the mesh density could explain the smaller distances with *Mesh1*.

We looked at the AP duration. For the 7 cases the reference APD90 varied between 225 and 285 ms. A difference was clearly visible between the endocardium and the epicardium. We were not able to reproduce this difference with *Mesh2*. However, APD90 were similar on the epicardium for both meshes. Our method tended to reconstruct maximal APD90s much higher than 285 ms, especially in cases 1, 2, and 7.

## 4. Discussion

We presented a new ECGI method designed to recover both the depolarization and the repolarization sequence, by solving a parameter identification problem. We hypothesized that this method would work better when both the endocardium and epicardium are included in the model, since important and physiologically relevant differences in both depolarization and repolarization timing exist between these surfaces. Therefore, we tested the method on two different heart meshes: the one a closed surface of the epicardium alone, and the other including both epicardium and endocardium. Tests were performed using *in silico* data for a sinus beat and six different ventricularly paced beats. Results were very similar for both meshes in 6 cases: all the characteristics we looked at were of the same good quality. The presence of the endocardium slightly improved the ATs on the epicardium. In contrast, for the RTs the effect of including the endocardium was variable. In two other cases (sinus rhythm, case 2, and septal pacing case 7), the reconstruction of AT with *Mesh1* was poor. In the sinus rhythm case, inclusion of the endocardium (*Mesh2*) improved the reconstruction substantially. This was the only case where endo-epicardial gradients, with respect to the total activation time, were significant. In all cases, the repolarization times were better reconstructed with the epicardium only.

We showed that our method was not sensitive to the initialization. Especially the choice for τ_out_ and τ_close_ did not impact the reconstruction of ATs, since these two parameters play a role only during the repolarization. Similarly, imposing global instead of distributed parameters will not worsen ATs reconstruction. The quality of the estimation of the Mitchell-Schaeffer parameters can only be seen through RTs and APD90 reconstructions. CC for RTs were smaller than the ones for ATs which may suggest that the reconstruction of τ_out_ and τ_close_ was less precise than ATs reconstruction. APD90 maps confirmed that, on a same case, we can overestimate as well as underestimate APD90 on large areas.

In general, our method underestimated AT delays between endocardium and epicardium (Figure 6). A possible explanation is that from the torso surface the two heart surfaces are too close to be seen separately. The endocardial activity is masked by the epicardial one, even in the case of endocardial pacing. The problems we solved, with *Mesh1* or *Mesh2*, were actually the same; we ended with similar results. It may also explain why we did not reconstruct APD differences between the epicardium and the endocardium.

Another possible explanation is the difference in density between the two meshes. We chose to have about the same number of nodes in each mesh, so that the difference in the number of parameters to identify could not alone explain the results. However, it implied that *Mesh2* was coarser than *Mesh1*. A test was made on a refined mesh of *Mesh2* (Figure 2, right). This third mesh had 1328 nodes and a density similar to the one of *Mesh1*. We solved the inverse problem on this mesh for the ventricular pacing case 1. The results we obtained were very similar to those with *Mesh2*: the CC for ATs was 0.79 (0.77 for *Mesh2*) and the average CC for the BSPM was 0.86 (0.9 for *Mesh2*). This test may suggest that the density of the mesh does not have an impact on the results.

We solved the inverse problem with a constant factor A over the whole heart. However, this factor (proportional to the amplitude of the AP) may not be constant, e.g., in the case of ischemia. We attempted to consider a distributed factor, more relevant from a physiological point of view. In that case the method was not converging, or converged to both positive and negative amplitudes.

So far we did not add noise to the testing data. Even if the models to create the data and to solve the inverse problem are different, it would be helpful to assess the robustness of the method.

Validation data were created from a volumetric heart mesh with a much higher density than *Mesh1* and *Mesh2*. The reference values (AT, RT) were the values on the mesh nodes. In contrast, the inverse problem on a surface leads to values that contain information averaged over a considerable volume. This may explain why the delays between reconstructed ATs were smaller than the delays between the reference ATs.

### 4.1. Comparison With Other Methods

Currently, most ECGI methods are based on a Laplace problem for the potential in the torso. Using the MFS (Wang and Rudy, 2006) or boundary-element models (Sapp et al., 2012; Bear et al., 2018) these methods reconstruct instantaneous potential patterns on the surface of the heart. These methods use Tikhonov or similar forms of regularization to counter the ill-posedness of this problem. This form of regularization leads to smooth solutions for the potential distribution, while the actual pattern, especially in case of an activation wavefront, is characterized by steep gradients. This leads to unrealistic solutions for the activation pattern, featuring large areas that appear to be activated nearly simultaneously, separated by artefactual lines of conduction block (Duchateau et al., 2017; Ravon et al., 2017). Various methods have been proposed to counter this effect, e.g., by reconstructing AT maps from local delays estimated from the whole signal morphology (Duchateau et al., 2017) or by simply smoothing the activation map (Bear et al., 2018). The latter method claims that it does not wipe out true block lines, as well as the artefactual ones, without any validation yet. The method that we proposed here does not require such postprocessing. It imposes a predefined action potential waveform, parameterized in terms of AT and parameters of the Mitchell-Schaeffer model, and does not require further regularization. We have previously shown that our method leads to more realistic activation maps than the MFS (Ravon et al., 2017). In the larger sample of this study we also did not observe the clustering of AT that is typical for MFS methods.

A similar parameter optimization approach, also in terms of endocardial and epicardial AT and RT, was used by van Dam et al. (2009). In contrast to our method it still relied on a (Laplacian) regularization of the AT field, and ahead of the parameter estimation phase it performed an initial estimate based on an exhaustive search. On the other hand, it used a more realistic volume conductor model that took the boundedness and inhomogeneity of the torso into account. Unlike our method they showed that the choice of the initial estimates had an impact on the quality of the inverse procedure. This importance had also been reported by Potyagaylo et al. (2016) and Erem et al. (2014).

Others have worked on the impact of the endocardium in the case of atrial fibrillation Schuler et al. (2017). Considering that atria are very thin, they imposed similar TMP values on the epicardium and the endocardium. Due to the greater thickness of the ventricles, this hypothesis would not be suitable in our study. In a previous study (Potyagaylo et al., 2014) the same group proposed a local regularization of the two surfaces to localize ectopic beats. The regularization parameter can differ between the endocardium and the epicardium. It was a way to better distinguish endocardial events from epicardial events. This approach might be applicable in our case with two different factors A.

### 4.2. Conclusion

Our parameter optimization method reconstructs accurate activation times and, to a lesser extent, repolarization times. In some cases inclusion of the endocardium in the solution helps to improve the reconstruction of activation times, while in general it does not improve the reconstruction of repolarization times.

## Author Contributions

All authors have made substantial contributions to this study. GR designed the study, implemented the algorithms, analyzed, and interpreted the results, and drafted the manuscript. YC and RD helped conceive the study, provided feedback about the implementation of the methods and the interpretation of the results, and revised the manuscript. MP provided the validation data and feedback about the results, and revised the manuscript.

### Conflict of Interest Statement

The authors declare that the research was conducted in the absence of any commercial or financial relationships that could be construed as a potential conflict of interest. The handling editor and reviewer LW declared their involvement as co-editors in the Research Topic, and confirm the absence of any other collaboration.
