# Supplementary figures and images for: Impact of the Endocardium in a Parameter Optimization to Solve the Inverse Problem of Electrocardiography
Source: Front Physiol. 2019 Jan 22;9:1946. doi: 10.3389/fphys.2018.01946 (PMC6349712; doi:10.3389/fphys.2018.01946)

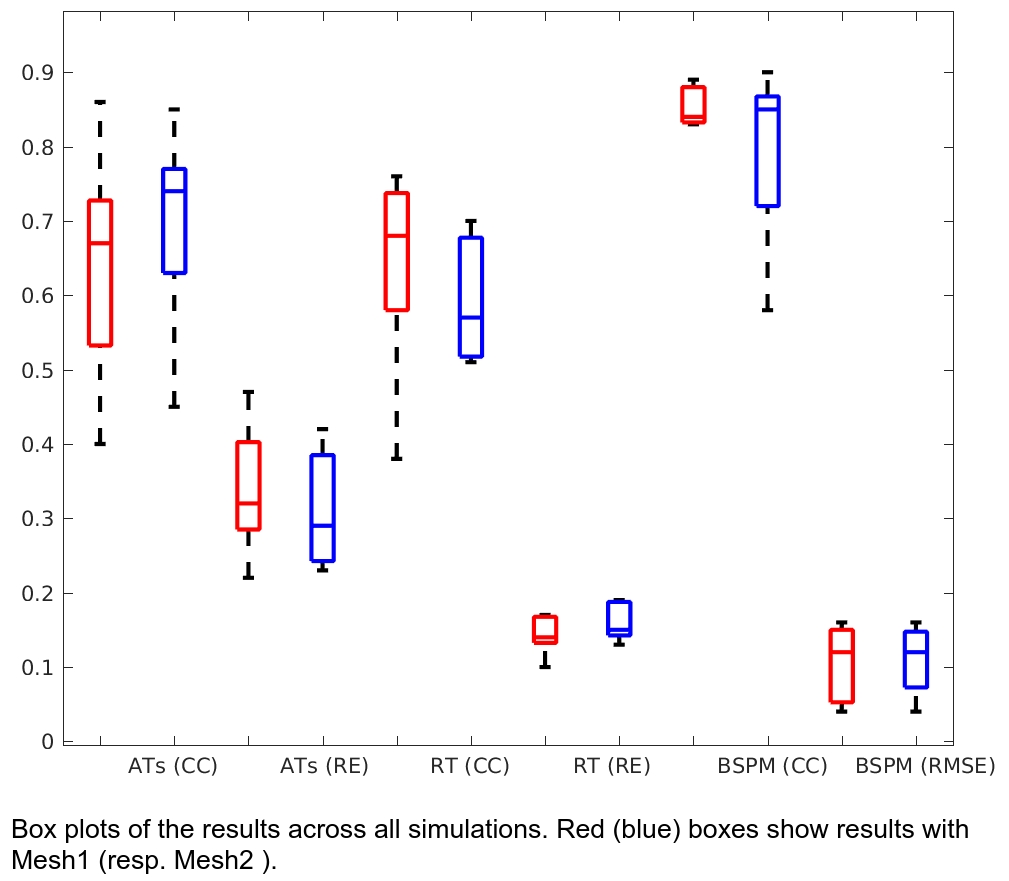

Supplement: Supplementary file 1 [file Image_1.JPEG]

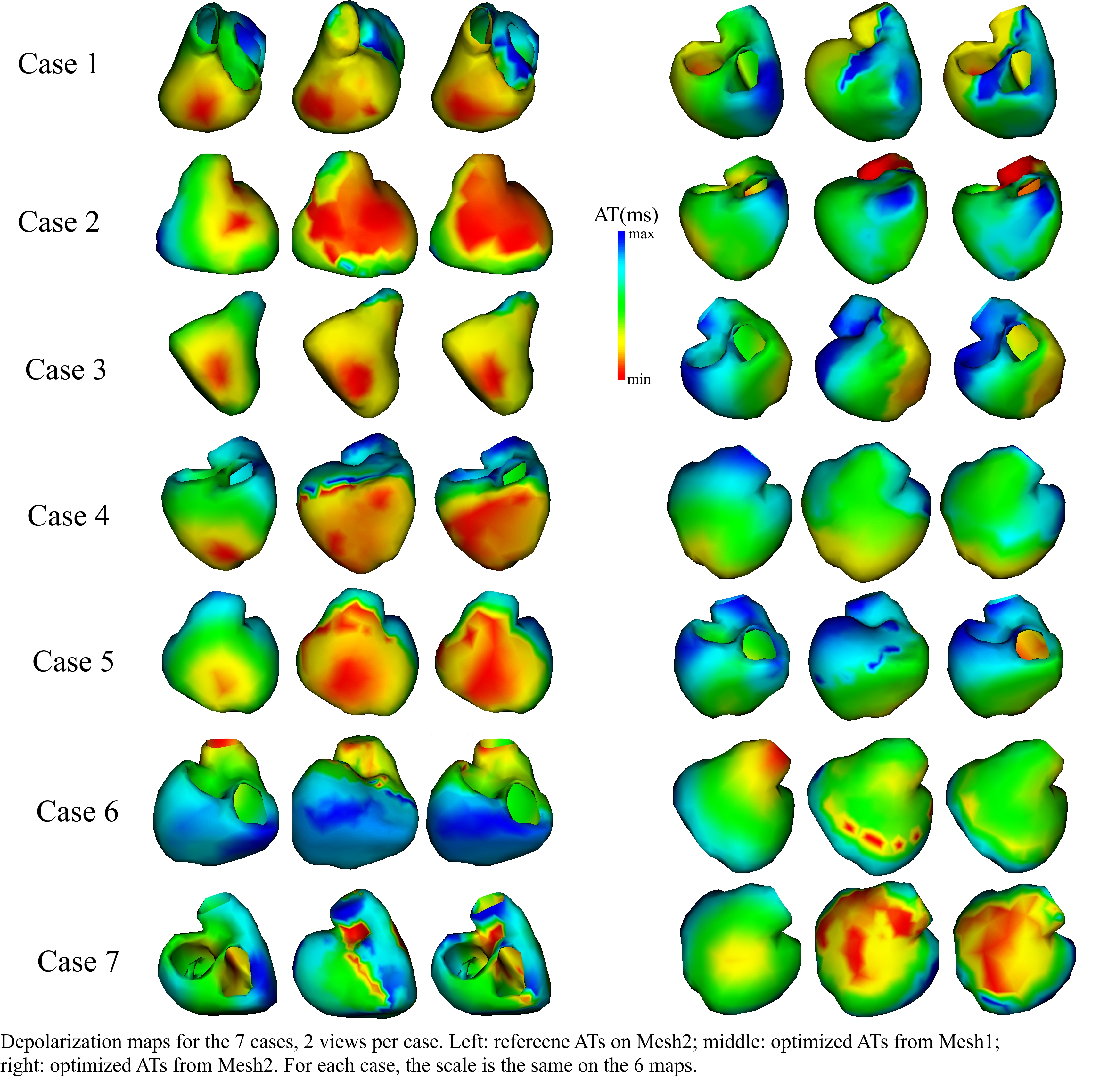

Supplement: Supplementary file 2 [file Image_2.JPEG]

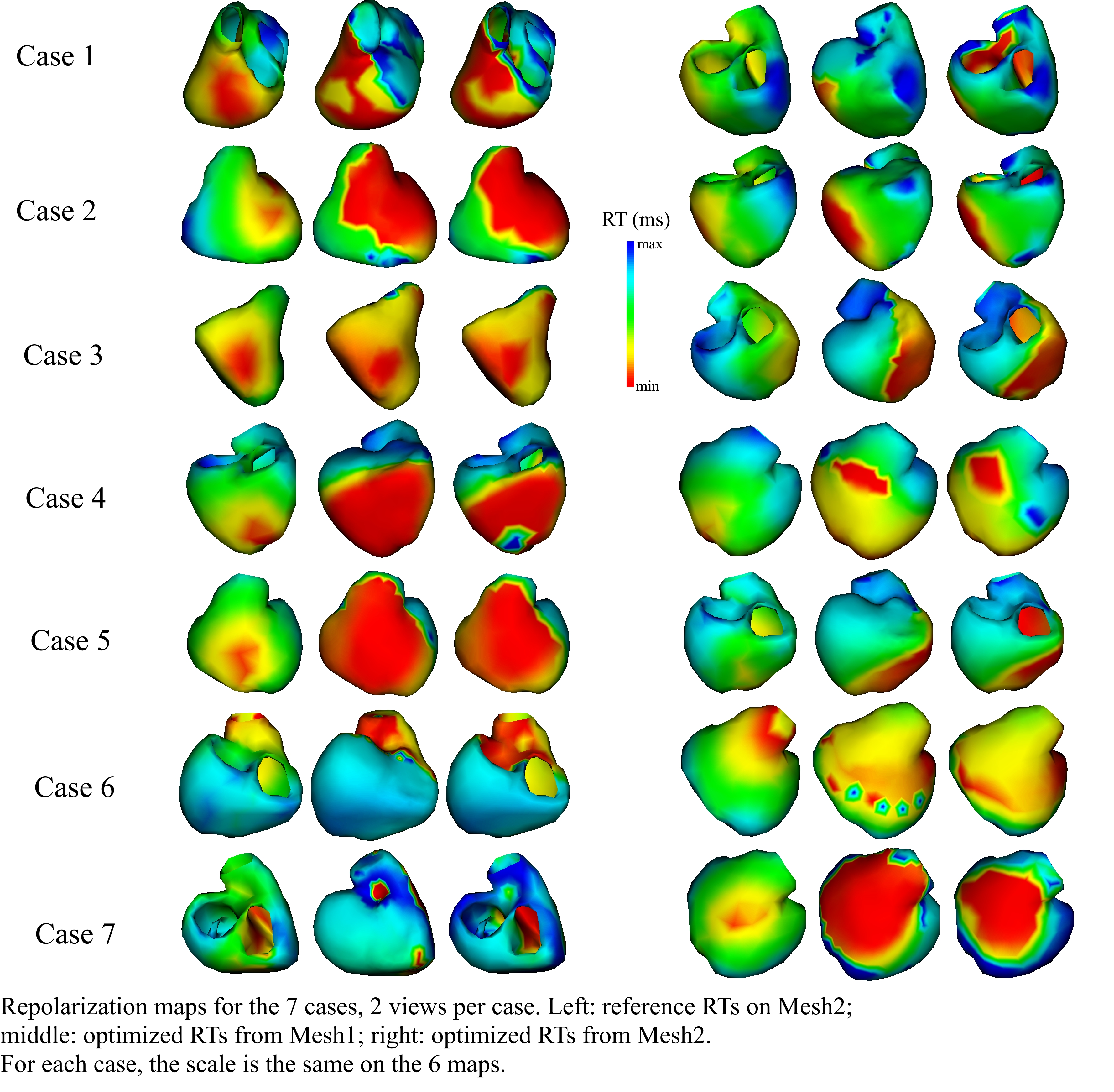

Supplement: Supplementary file 3 [file Image_3.JPEG]

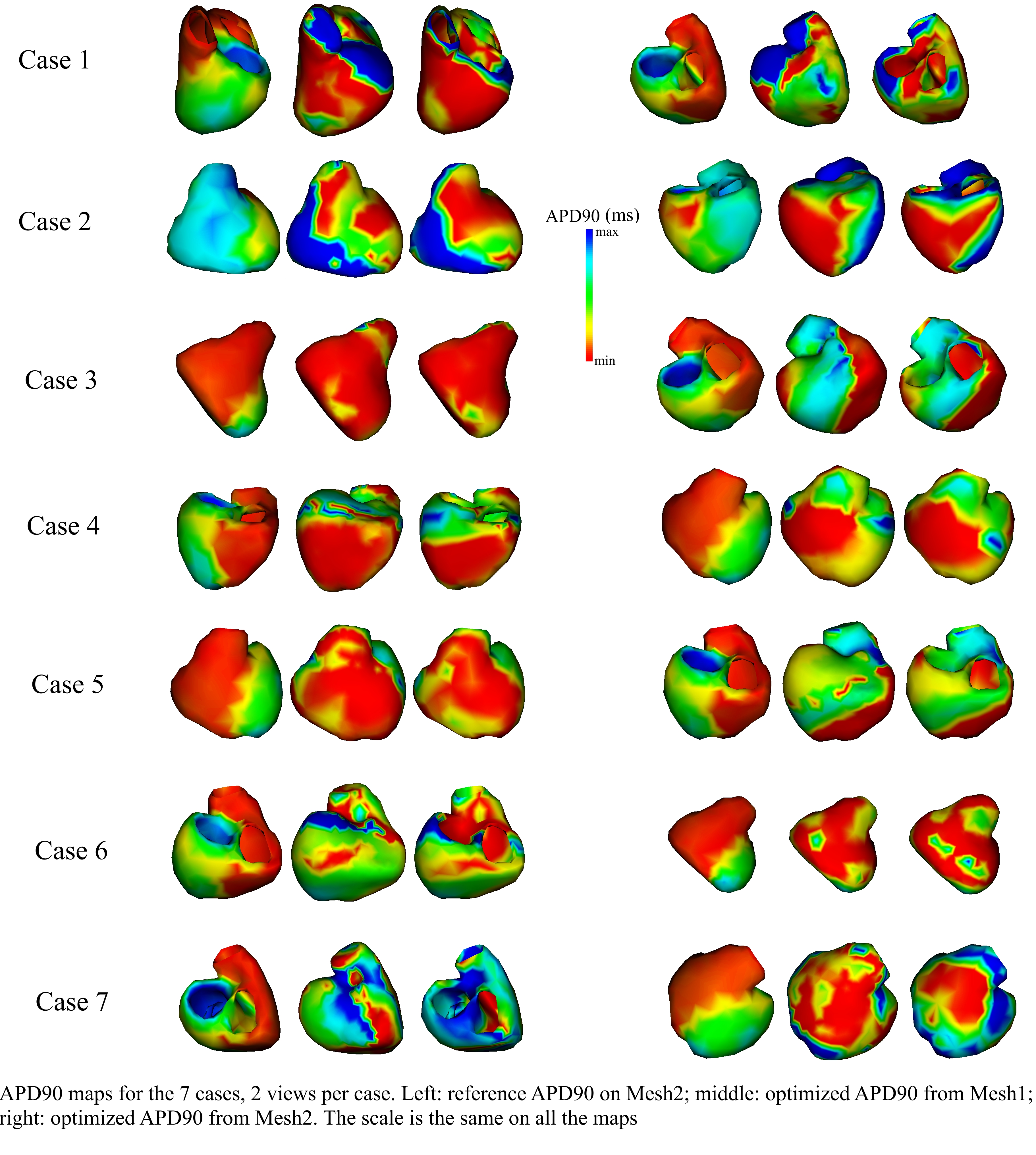

Supplement: Supplementary file 4 [file Image_4.JPEG]

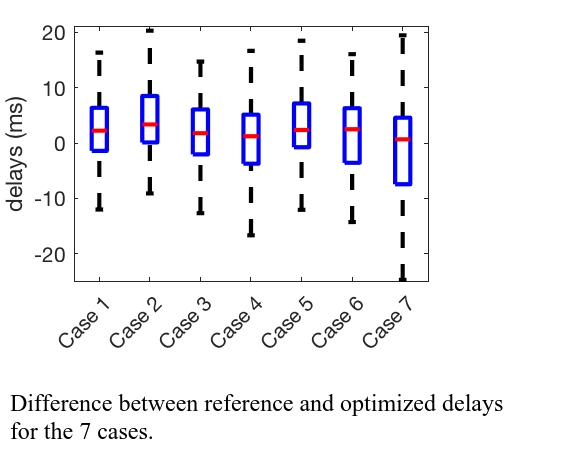

Supplement: Supplementary file 5 [file Image_5.JPEG]
